# Acupuncture for diabetic nephropathy: mechanisms, clinical evidence, and future perspectives

**DOI:** 10.3389/fendo.2025.1335495

**Published:** 2025-11-13

**Authors:** Jing Yue, Jinhao Guo, Xinru Wang, Yifan Kong, Lingwei Song, Yuanxiang Liu, Yipeng Liu, Ji-Guo Yang

**Affiliations:** 1 College of Acupuncture and Massage, Shandong University of Traditional Chinese Medicine, Jinan, Shandong, China; 2 Department of Traditional Chinese Medicine, Shandong Second Provincial General Hospital, Jinan, Shandong, China; 3 Shandong College of Traditional Chinese Medicine, Yantai, Shandong, China; 4 The First Clinical Medical College, Shandong University of Traditional Chinese Medicine, Jinan, Shandong, China; 5 Department of Nephrology, The First Affiliated Hospital of Shandong First Medical University and Shandong Provincial Qianfoshan Hospital, Jinan, Shandong, China; 6 Department of Nephrology, Shandong Provincial Qianfoshan Hospital, Shandong University, Jinan, Shandong, China; 7 Nephrology Research Institute of Shandong Province, Jinan, Shandong, China

**Keywords:** diabetic nephropathy, etiology and pathogenesis, acupoint, acupuncture treatment, electroacupuncture, Chinese medicine

## Abstract

Diabetic nephropathy (DN) remains a leading cause of end-stage renal disease despite guideline-based therapy. Acupuncture has been explored as an adjunct or alternative approach. We reviewed preclinical and clinical studies (2010–2025) on acupuncture for DN, summarizing mechanisms, intervention models (acupuncture alone; with Chinese medicine; with Western medicine; triple therapy), renal outcomes, and safety. Across animal and human data, acupuncture modulates immune–inflammatory and metabolic pathways—including HMGB1/NLRP3/NF-κB, SIRT1/AMPK/PGC-1α, eNOS–NO–cGMP, and autophagy (ULK1–Beclin-1–LC3)—enhances antioxidant defenses (SOD↑, MDA/8-OHdG↓), protects podocytes, and improves microcirculation. Clinically, it is associated with reductions in proteinuria (24-h UP, UACR/UAER), improvements in renal function (Scr, BUN, eGFR), and better metabolic control and symptoms. Combined regimens (with Chinese or Western medicines) tend to yield faster or broader benefits, with no serious adverse events reported in the included studies. Evidence quality is limited by small sample sizes, single-center designs, short follow-up, heterogeneous endpoints, and incomplete safety reporting. Acupuncture shows multi-target, complementary effects for DN and may be integrated with standard care. High-quality, multicenter randomized controlled trials with standardized endpoints (e.g., proteinuria, eGFR slope), robust safety monitoring, and embedded mechanistic assessments are warranted.

## 1 Introduction

Diabetic nephropathy (DN) is one of the most common and serious complications of diabetes and a leading cause of end-stage renal disease (ESRD) (1–3) Globally, about 537 million adults (10.5%) have diabetes, and this number is expected to reach 783 million by 2045. In China, 141 million people have diabetes, with 20%–40% affected by DN (4). DN is often asymptomatic in its early stage but can progress to proteinuria, hypertension, and edema (5–7). Current treatments include lifestyle modification, strict glycemic control, regulation of blood pressure and lipid levels (8, 9), and the use of renoprotective agents such as angiotensin-converting enzyme inhibitors (ACEIs), angiotensin II receptor blockers (ARBs), and sodium-glucose cotransporter 2 inhibitors (SGLT2is) (10). Although these therapies may delay disease progression, they cannot fully prevent renal decline, and drugs such as ACEIs and ARBs may increase the risk of irreversible kidney injury or diabetic ketoacidosis (DKA) (11–13). Therefore, DN remains a significant burden for both patients and society, underscoring the urgent need for novel adjuvant or alternative treatments. A growing number of emerging therapies are currently under development (14, 15).

Among complementary options, traditional Chinese medicine (TCM) has drawn growing attention, with acupuncture—one of its core therapies—providing a long-standing basis for DN intervention. With a history of over 3,000 years of application in China, acupuncture has been extensively utilized in the treatment of diabetes and chronic kidney diseases (16–20). Based on meridian and acupoint theory, acupuncture stimulates specific points to produce propagated sensation along the meridians (PSM), thereby regulating organ function and balancing qi and blood (21–25). Clinical practice suggests that acupuncture offers multi-target regulation, holistic effects, reliable efficacy, minimal side effects, and good patient compliance. Acupuncture has been widely used in conditions such as chronic pain, stroke rehabilitation, insomnia, gastrointestinal disorders, and hypertension, with favorable outcomes (26–30). These experiences provide an important basis for the prevention and treatment of metabolic diseases such as diabetes with acupuncture (31, 32).

Recent studies on acupuncture for DN have increased, with evidence from animal and clinical research suggesting benefits such as reduced proteinuria, suppressed inflammation, improved glucose and lipid metabolism, enhanced renal hemodynamics, and protection of podocytes (33–36). Compared with conventional drugs, acupuncture appears safer, well tolerated, and complementary to standard therapy, showing potential value in long-term and integrative care. However, most studies are limited by small sample sizes, single-center designs, or methodological weaknesses, and the quality of evidence remains uneven, reducing confidence in its clinical application (18).

To improve clarity, we added a methodology section. We searched PubMed, Web of Science, and CNKI from 2010 to 2025 using terms such as “diabetic nephropathy,” “acupuncture,” “traditional Chinese medicine,” and “renal protection,” and also screened references through snowballing. Studies were included if they reported renal outcomes (24-h UP, UACR/UAER, Scr, eGFR) and/or mechanistic indicators (eNOS/NO, HMGB1/NLRP3/NF-κB, SIRT1/PGC-1α, nephrin/podocin). We excluded case reports, reviews without original data, and studies lacking methodological clarity or relevance. This review is presented as a narrative review, with study inclusion based on relevance and methodological adequacy. As a narrative review, we did not perform formal risk-of-bias scoring; instead, inclusion was based on relevance to our conceptual framework, methodological adequacy, and representativeness. Priority was given to recent studies and those reporting renal outcomes (proteinuria, Scr, eGFR) or mechanistic indicators (oxidative stress, autophagy, podocyte markers).

Unlike earlier narrative reviews, this article develops a testable pathogenesis–mechanism–indicator–prescription framework that bridges Western medicine and traditional Chinese medicine (TCM). By focusing on key acupoints (ST36, SP6, and BL23) and root–branch prescriptions, we integrate molecular pathways (HMGB1/NLRP3/NF-κB, SIRT1/PGC-1α, eNOS/NO, and autophagy markers) with renal outcomes (proteinuria, Scr, and eGFR). This work further combines clinical and mechanistic evidence to construct a multi-level chain of evidence, highlight the therapeutic potential of acupuncture, and propose future directions for research and clinical translation in diabetic nephropathy.

## 2 Pathogenesis of diabetic nephropathy: integrating Western and TCM insights

### 2.1 Pathogenesis of Western medicine

The pathogenesis of diabetic nephropathy (DN) is complex, involving immune and inflammatory responses, metabolic disorders, renal hemodynamic abnormalities, oxidative stress, dysregulated autophagy, and mechanisms related to epigenetics and the gut–kidney axis (37). Hemodynamic and metabolic disturbances often initiate disease by activating multiple signaling pathways in renal tissue (38). Increasing evidence suggests that abnormal immune and inflammatory responses are central drivers of DN. Under hyperglycemia, macrophages and T cells infiltrate the glomeruli and tubulointerstitium, accompanied by persistent upregulation of pro-inflammatory cytokines (TNF-α, IL-1β, IL-6) and chemokines (MCP-1, CXCL family), leading to chronic inflammation (39). A key feature is macrophage polarization imbalance: M1 macrophages release pro-inflammatory mediators, including reactive oxygen species (ROS), that aggravate injury, whereas reduced M2 polarization limits repair and anti-inflammatory activity. Crosstalk between macrophages and podocytes further promotes disease progression. Macrophages can suppress podocyte cytoskeletal proteins (nephrin, podocin) through exosomal miRNAs and inflammatory mediators, disrupting the filtration barrier, while injured podocytes release damage-associated molecular patterns (DAMPs) that reactivate macrophages, forming a self-amplifying pathological loop (40).

Oxidative stress is a central mechanism in DN progression. Hyperglycemia and metabolic disturbances induce excess ROS, which directly damage podocytes, mesangial cells, and endothelial cells, disrupting the filtration barrier and causing proteinuria (41). ROS also activate other pathogenic pathways that, in turn, amplify injury through oxidative stress (42). In addition, oxidative stress acts synergistically with metabolic and hemodynamic abnormalities (43). Ansari et al. reported that inflammation and fibrosis form a positive feedback loop: pro-inflammatory factor upregulation and TGF-β activation promote fibrosis, while fibrosis progression further aggravates inflammation, creating a vicious cycle of “inflammation–fibrosis–injury” (44). Overall, oxidative stress—together with inflammation, fibrosis, and programmed cell death—drives DN from functional impairment to structural damage.

Beyond immune responses and oxidative stress, the gut–kidney axis, epigenetics, and autophagy have gained attention in recent years. Gut microbiota imbalance may contribute to DN, as metabolites such as short-chain fatty acids and indole derivatives can alter the renal microenvironment through immune and metabolic pathways, suggesting a regulatory role upstream of inflammation and metabolism (45). Epigenetic mechanisms, including DNA methylation, histone modification, and non-coding RNAs (miRNAs, lncRNAs), sustain inflammatory and fibrotic activation and may explain variability in disease progression and treatment response. Exosomes, as carriers of epigenetic information, transfer pathogenic molecules between renal cells and amplify injury signals (46). Autophagy is also abnormal in DN: reduced activity impairs clearance of damaged mitochondria and protein aggregates, while excessive activation under high-glucose and high-lipid conditions may trigger apoptosis and worsen injury. Therapeutic modulation of autophagy is considered a promising strategy for kidney protection (47). Genetic epidemiology has also added evidence for immune and inflammatory mechanisms in DN. A bidirectional Mendelian randomization study found that the causal associations of some immune cell subsets with DN were weak, with most losing significance after multiple testing.

The pathogenesis of DN is multifactorial, involving immune dysregulation, oxidative stress, and metabolic and hemodynamic disturbances. Traditional Chinese medicine (TCM) regards kidney-qi deficiency as the root, with dampness, heat, stasis, and phlegm as key patterns. Notably, kidney-qi deficiency parallels immune dysfunction, blood stasis reflects microcirculatory disturbance, dampness–heat corresponds to metabolic and oxidative stress, and phlegm aligns with metabolic imbalance and impaired autophagy. Beyond these overlaps, TCM provides a holistic framework that may compensate for gaps in biomedical models, particularly in integrating systemic dysfunctions and guiding individualized care.

### 2.2 Pathogenesis of TCM

Building on these biomedical insights and observed parallels, traditional Chinese medicine (TCM) provides a complementary framework to explain the pathogenesis of diabetic nephropathy (DN). Although DN primarily involves the kidney, TCM holds that it can also affect the spleen, stomach, liver, and other viscera. There are four main aspects of the etiology, as follows. First, kidney-qi deficiency. The kidney is regarded as the congenital foundation, gathering the essence of the five zang organs and six fu organs. Kidney essence supports various functions of the viscera under the regulation of kidney-qi, such as the production and excretion of urine. If the natural endowment is insufficient, the five viscera will be weak, and instability of kidney-qi will further lead to leakage of essence and kidney disease (48). Therefore, deficiency of natural endowment and weakness of the five viscera are precipitating factors for disease onset and influencing factors for disease progression. TCM believes that the kidney is mainly responsible for water metabolism, and kidney-qi deficiency is one of the major causes of DN. Long-term hyperglycemia and excessive kidney load can lead to kidney-qi depletion, chronic kidney injury, and kidney dysfunction.

Second, blood stasis blocking the collaterals. Abnormal blood glucose levels can trigger increased blood viscosity and impaired blood circulation, resulting in insufficient blood supply to the kidney and eventually leading to the formation of blood stasis blocking the collaterals (49). Third, dampness–heat in the spleen and stomach. TCM holds that the spleen and stomach are the main organs responsible for water metabolism. Long-term improper diet together with spleen–stomach dysfunction can lead to dampness–heat. Patients with diabetes often have poor dietary habits and spleen–stomach weakness, resulting in heat accumulation and eventually leading to DN (50). Fourth, liver–kidney disharmony. TCM believes that the liver and kidney govern free coursing and excretion, respectively, influencing the occurrence and development of DN. Both liver-qi stagnation and liver–kidney yin deficiency may contribute to impaired renal function (51).

In summary, kidney-qi deficiency corresponds to the principle of replenishing kidney-qi; blood stasis blocking the collaterals can be treated by promoting blood circulation and removing stasis; dampness–heat in the spleen and stomach requires regulation of the spleen and stomach; and liver–kidney disharmony can be managed by harmonizing the liver and kidney. Such correspondence highlights the consistency between pathogenesis and treatment in TCM. With the purpose of restoring normal kidney function and effectively relieving clinical symptoms, the main concepts of TCM treatments include: (i) treatment based on pattern differentiation; (ii) consideration of seasonal, environmental, and constitutional factors; (iii) addressing both manifestations and root causes (biao–ben); (iv) regulating the spleen and stomach; (v) replenishing kidney-qi; (vi) promoting blood circulation to remove blood stasis; and (vii) regulating the liver and kidney.

In addition to these classical categories, modern scholars have proposed refined conceptual frameworks to explain the complex pathogenesis of DN in TCM. Tong et al. (52) identified deficiency, stasis, and damage as the core pathogenesis of DN, with the principle of treating both deficiency and excess as the guiding theory, while supplementing deficiency, promoting blood circulation, and warming yang to eliminate turbidity (wenyang xiezhuo) are regarded as the basic therapeutic methods. In addition, according to the emphasis on deficiency, stasis, and damage, Huangqi Jianzhong Decoction has been used as the main prescription. Blockage of meridians is considered to occur throughout the course of DN; thus, treatments should aim to promote blood circulation and remove meridian obstruction, with the basic use of Chuan Xiong (Rhizoma Chuanxiong), Yuan Hu (Rhizoma Corydalis), and Zhi Qiao (Fructus Aurantii), and the additional use of Shi Xiao San to remove stasis. At the later stage of DN, Wu Ling San and Dahuang Fuzi Decoction are often used to treat internal turbidity and toxicity. Long-term DN often stems from innate and acquired deficiencies, leading to spleen–kidney weakness. This causes qi deficiency and impaired transformation, resulting in pathological dampness, stasis, and phlegm that worsen the condition.

**Figure 1:**
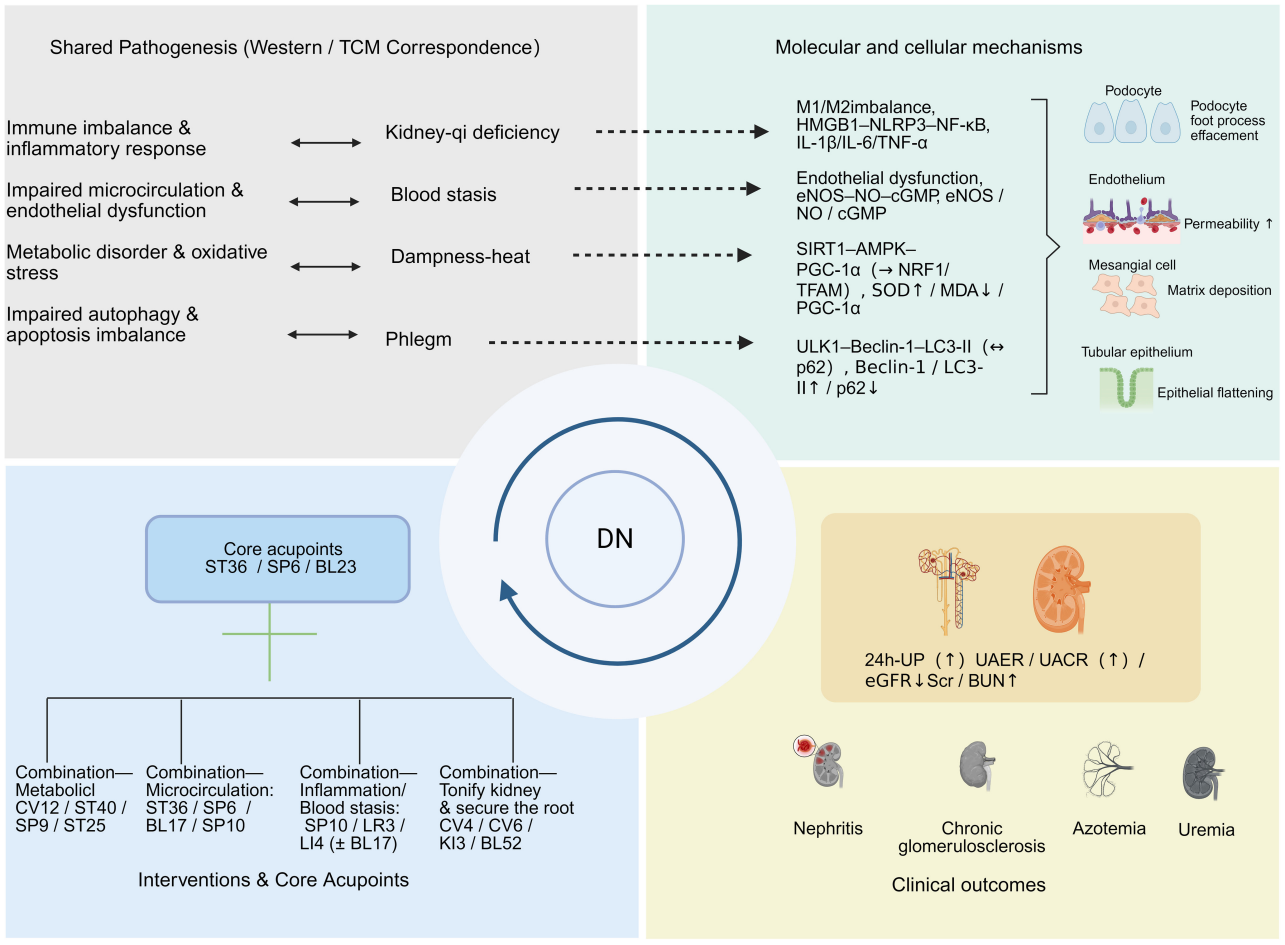
Integrated map of diabetic nephropathy (DN) pathogenesis, mechanisms, interventions, biomarkers, and clinical outcomes. Top left: Shared pathogenesis and TCM mapping—immune imbalance/inflammation ↔ kidney-qi deficiency; microcirculatory disturbance/endothelial dysfunction ↔ blood stasis; metabolic disorder/oxidative stress ↔ damp–heat; impaired autophagy and apoptosis imbalance ↔ phlegm (dashed arrows = putative links). Top right: Molecular and cellular mechanisms—(i) immune/inflammation: M1/M2 imbalance, HMGB1–NLRP3–NF-κB, IL-1β/IL-6/TNF-α; (ii) endothelium/microcirculation: eNOS–NO–cGMP; (iii) metabolism/oxidative stress: SIRT1–AMPK–PGC-1α → NRF1/TFAM, SOD ↑/MDA ↓; (iv) autophagy/apoptosis: ULK1–Beclin-1–LC3-II (↔ p62). Icons indicate podocyte foot process effacement, increased endothelial permeability, mesangial matrix deposition, and tubular epithelial flattening. Bottom left: Interventions and core acupoints—core: ST36/SP6/BL23; combinations: metabolic (CV12/ST40/SP9/ST25), microcirculation (ST36/SP6/BL17/SP10), inflammation–blood stasis (SP10/LR3/LI4 ± BL17), and tonify kidney and secure the root (CV4/CV6/KI3/BL52). Bottom right: Biomarkers and outcomes—24-h UP ↑, UAER/UACR ↑, eGFR ↓, Scr/BUN ↑, with downstream outcomes (nephritis, chronic glomerulosclerosis, azotemia, uremia). Arrows denote direction of change.

In summary, the pathogenesis of diabetic nephropathy (DN) involves immune and inflammatory responses, oxidative stress, metabolic disorders, hemodynamic abnormalities, and impaired autophagy. Acupuncture, a major therapy in traditional Chinese medicine (TCM), has been shown to act on these interconnected pathways: immunomodulation reduces inflammation, which in turn alleviates oxidative stress and protects podocytes; improved microcirculation enhances renal perfusion and helps mitigate fibrosis; and regulation of autophagy supports cellular homeostasis. Through these integrative effects, acupuncture may slow DN progression and complement standard therapies (**Figure 1**).

## 3 Integrative acupuncture and drug therapies for diabetic nephropathy

### 3.1 Acupuncture in diabetes: A foundation for DN research

Based on the theory of zang–fu organs and meridians, acupuncture for diabetic nephropathy (DN) is guided by syndrome differentiation and follows the principle of “tonifying the kidney and spleen, promoting blood circulation, and unblocking the collaterals,” reflecting its holistic and multi-target regulatory effects (53). In clinical practice, commonly used acupoints are mainly located on the Foot Taiyang Bladder Meridian, Foot Yangming Stomach Meridian, Conception Vessel, Foot Taiyin Spleen Meridian, and Foot Shaoyin Kidney Meridian (54). Representative points include Zusanli (ST36), Shenshu (BL23), Sanyinjiao (SP6), and Taixi (KI3). The Bladder Meridian runs along both sides of the spine and extends to the lower limbs, with its Back-Shu points distributed across the lumbar and dorsal regions, allowing regulation of internal organ function through qi convergence (55, 56). The Conception Vessel, regarded as the “Sea of Yin Vessels,” governs yin-qi, while the Governor Vessel, the “Sea of Yang Vessels,” governs yang-qi; together, they interact to maintain yin–yang balance (57). This zang–fu–meridian framework provides a traditional theoretical basis for the clinical use of acupuncture in DN.

Modern studies show that acupuncture improves the pathological processes of diabetes and its complications—including DN and diabetic peripheral neuropathy—through multiple pathways and targets. First, it regulates the insulin signaling pathway by upregulating IRS-1, IRS-2, PI3K, Akt, and GLUT4, and by activating AMPK and PGC-1α signaling, thereby enhancing insulin sensitivity, mitochondrial function, and energy metabolism (58). Second, it suppresses the NF-κB and JAK/STAT pathways, reduces inflammatory cytokines such as TNF-α and IL-6, and alleviates inflammatory injury (59). Third, acupuncture increases the activity of antioxidant enzymes such as superoxide dismutase (SOD) and glutathione peroxidase (GSH-Px), while lowering malondialdehyde (MDA) levels, thereby mitigating oxidative stress; it also improves microcirculation and renal hemodynamics, reducing ischemic and hypoxic injury (60). In addition, evidence suggests that acupuncture may regulate autophagy through pathways including SIRT1 and AMPK/mTOR, promote the clearance of damaged mitochondria and abnormal proteins, and contribute to the regulation of inflammation and fibrosis (61).

It is important to note that these effects are interconnected rather than independent: improved metabolism reduces oxidative stress, while lower oxidative stress suppresses inflammatory responses; inflammation control supports blood flow and cellular recovery; improved hemodynamics relieve ischemic stress; and, together with regulation of autophagy and apoptosis, these processes help maintain cellular and systemic homeostasis. In summary, acupuncture modulates the pathological loop of “metabolism–oxidative stress–inflammation–hemodynamics–cellular homeostasis,” thereby exerting holistic, multi-target effects. As DN is one of the most common and severe microvascular complications of diabetes, sharing key pathogenic mechanisms with diabetes itself, acupuncture has been increasingly applied in DN prevention and treatment, either as a stand-alone therapy or in combination with Western medicine.

### 3.2 Acupuncture therapy alone

Zhang et al. (33) showed in a mouse model that electroacupuncture (EA) at Yishu (EX-B3), Shenshu (BL23), Zusanli (ST36), and Sanyinjiao (SP6) reduced IL-1β and IL-6 levels and inhibited the HMGB1/NLRP3/NF-κB pathway. These findings suggest that EA may help slow the progression of diabetic nephropathy (DN) by suppressing inflammasome activation and blocking the feedback loop between inflammation and oxidative stress. Huang et al. (62) reported in a rat model that EA at BL23 and ST36 reduced 24-h UP, proteinuria, and blood urea nitrogen, while upregulating autophagy-related proteins such as Beclin-1 and nephrin, indicating a link between enhanced autophagy and renal protection. Wang et al. (63) reported in a randomized clinical trial that EA at Biaoben acupoints—Zhongwan (CV12), Fenglong (ST40), Xuehai (SP10), Taichong (LR3), Guanyuan (RN4), and ST36—produced greater improvements in renal function and microcirculation in patients with early DN compared with conventional acupoints, and this effect was accompanied by increased eNOS/NO levels. In an animal study, Wang et al. (64)demonstrated that treatment at Biaoben acupoints—ST36, Guanyuan (CV4), ST40, and CV12—enhanced renal FoxO1 and PGC-1α expression, increased SOD activity, and reduced MDA and ROS levels, indicating that EA may restore antioxidant capacity through activation of the FoxO1/PGC-1α pathway and thereby attenuate oxidative stress. Chen et al. (65) further showed in rats that acupuncture at ST40, CV12, ST36, and CV4 improved mitochondrial function via the SIRT1/PGC-1α pathway and attenuated renal injury. Li et al. (66) reported that Tiaolipiwei acupuncture reduced 24-h UP and mitigated podocyte damage, supporting a filtration-barrier–stabilizing effect. Wang et al. (67)found in type 2 diabetic rats that EA pretreatment alleviated proteinuria by modulating the PTEN/PI3K/Akt signaling pathway, upregulating nephrin and podocin, and improving the balance between podocyte apoptosis and survival. In addition, a recent review highlighted that acupuncture at ST36 alone exerts strong anti-inflammatory effects, improves lung function, reduces renal injury markers (blood urea nitrogen and creatinine), and provides multi-organ protection for the heart, liver, kidney, and intestine (68). See **Table 1** for a summary of mechanism studies on different acupoints in improving DN.

**Table 1:** Summary of mechanism studies on different acupoints in improving DN.

Acupoints	Channel tropism	Presentation	Targets	Model	Evidence strength and safety
Yishu (EX-B3), Shenshu (BL23), Zusanli (ST36), Sanyinjiao (SP6)	Extra-point, Pangguang (bladder) meridian, Wei (stomach) meridian, Pi (spleen) meridian	Scr ↓	Inhibit HMGB1/NLRP3/ NF-κB pathway IL-1β、IL-6↓	Mice	Moderate (●●○); preclinical evidence; limited clinical; safe(39)
Shenshu (BL23), Zusanli (ST36)	Extra-point, Pangguang (bladder) meridian, Wei (stomach) meridian, Pi (spleen) meridian Pangguang (bladder) meridian, Wei (stomach) meridian	24h-UV, 24h-UP, BUN↓	Beclin-1 ↑,LC3-II ↑,p62 ↓	Rats	Moderate (●●○); preclinical evidence; limited clinical; safe (40)
Zhongwan (CV12), Fenglong (ST40), Xuehai (SP10), Taichong (LR3), Guanyuan (RN4), Zusanli (ST36)	Conception vessel, Wei (stomach) meridian, Gan (liver) meridian	UAER, Scr, BUN, CysC↓	eNOS, NO↑	Human	High(●●●); clinical RCT of harmonizing spleen-stomach in early DN; Safe(41)
Quchi (LI11), Zhigou (TE6), Hegu (LI4), Xuehai (SP10), Zusanli (ST36), Yinlingquan (SP9), Fenglong (ST40), Diji (SP8), Sanyinjiao (SP6), Taichong (LR3), Tianshu (ST25), Gaohuang (BL43), Shenshu (BL23), Baihuanshu (BL30), Zhongwan (CV12), Zhongji (CV3)	Dachang (large intestine) meridian, Sanjiao meridian, Pi (spleen) meridian, Wei (stomach) meridian, Gan (liver) meridian, Pangguang (bladder) meridian, conception vessel	Scr, BUN, ALB, UAER↓	MCP-1 expression↓	Human	Moderate (●●○); clinical RCT of harmonizing spleen-stomach in early DN; Safe(42)
Zusanli (ST36), Guanyuan (CV4), Fenglong (ST40), Zhongwan (CV12)	Wei (stomach) meridian, conception vessel	FBG, Scr, 24 h-UP↓	SIRT 1, PGC-1α expression↑	Rats	Moderate (●●○); preclinical evidence; limited clinical; safe(43)
Quchi (LI11), Zusanli (ST36), Diji (SP8), Zhongwan (CV12), Yinlingquan (SP9), Hegu (LI4), Sanyinjiao (SP6), Baichongwo (EX-LE3), Fenglong (ST40), Taichong (LR3)	Dachang (Large Intestine) meridian, Wei (stomach) meridian, Pi (spleen) meridian, conception vessel, Extra-point, Gan (liver) meridian	24 h-UP↓	PCX, CD2AP, nephrin↑ desmin↓	Rats	Moderate (●●○); preclinical evidence; limited clinical; safe(44)

Evidence strength grading (animal and clinical): High (●●●) = at least one clinical randomized/controlled study plus consistent preclinical support; Moderate (●●○) = small clinical or single-center RCT observational, or strong preclinical with limited clinical evidence; Limited (●○○) = mainly preclinical. Safety: no serious adverse events reported in included studies.

“↑” denotes an increase (higher value) and “↓” denotes a decrease (lower value) relative to the comparator (control/baseline).

In summary, both animal and clinical studies have shown that acupuncture alone is effective, with mechanisms involving anti-inflammatory activity, antioxidation, autophagy regulation, podocyte protection, and improvement of microcirculation. Core acupoints are mostly ST36, BL23, and SP6, often combined with “root–branch” point prescriptions (abdominal Conception Vessel points plus lower limb meridian points). Clinical evidence indicates that acupuncture can reduce proteinuria and improve renal function; however, its efficacy is limited by small sample sizes, short follow-up periods, and methodological weaknesses.

It is noteworthy that although acupuncture alone demonstrates benefits through multiple mechanisms, it remains insufficient in improving multidimensional outcomes and achieving consistent symptom relief. This provides a foundation for further research on integrated interventions such as “acupuncture combined with herbal medicine”.

### 3.3 Acupuncture combining with Chinese patent medicines

Current evidence indicates that combining acupuncture with herbal medicine is more effective than acupuncture alone in improving proteinuria and clinical symptoms. Xu et al. (69, 70) reported that in patients with diabetic nephropathy (DN) of the spleen–kidney yang deficiency pattern, warm needling at BL23, Qihai (CV6), Yishe (BL49), Shangjuxu (ST37), Huantiao (GB30), ST36, Taixi (KI3), and LR3, together with the Bupi Yishen Decoction, significantly improved poor appetite, frequent urination, fatigue, and soreness and weakness of the waist and knees, while also reducing 24-h UP, serum creatinine (Scr), and serum albumin. Hu et al. (71) reported the clinical efficacy of combined Yishen Huoxue Xiezhuo Decoction and Lu’s acupuncture in treating phase III DN. Their results showed consistent reductions in urinary protein and multiple biochemical parameters compared with baseline or standard care. Tang et al. (72) reported in a randomized controlled trial that the Yiqi Yangyin Huoxue formula combined with acupuncture lowered IL-1β, IL-17, and serum amyloid A (SAA) levels, and improved blood glucose and renal function, indicating a synergistic effect with core acupoints such as ST36, SP6, and BL23.

The combined use of acupuncture and herbal medicine follows the principles of “treating both root and branch, harmonizing zang–fu organs, and unblocking the collaterals to remove stasis.” Commonly used acupoints include ST36, SP6, and BL23, often combined with CV12, CV4/CV6, ST25, SP10, ST40, and LR3 to support spleen–stomach function, regulate the liver and kidney, and eliminate phlegm and stasis. Herbal prescriptions are generally based on tonifying qi, strengthening the spleen, warming kidney yang, promoting blood circulation, and draining dampness. Compared with acupuncture alone, the combination shows more consistent multidomain improvements—proteinuria and Scr/BUN decrease, glucose and lipid profiles improve, and pro-inflammatory cytokines (e.g., IL-1β, IL-17, SAA) decline—suggesting additive effects on symptoms and metabolism.

In summary, acupuncture combined with herbal medicine demonstrates the advantage of “treating both root and branch” and shows better overall efficacy than acupuncture alone. The incremental mechanisms may include stronger anti-inflammatory and antioxidant effects, more pronounced hemodynamic regulation, and additional support for the filtration barrier in syndrome-defined patients. However, most studies are limited by small sample sizes, single-center designs, short follow-up periods, and inadequate reporting of randomization, blinding, and safety, while the independent effects of acupuncture and herbal medicine remain unclear. Future multicenter, large-sample randomized controlled trials with stratified designs are needed to clarify their synergistic effects and identify optimal treatment strategies.

### 3.4 Acupuncture combining with Western medications

Chu et al. (73)compared acupuncture plus conventional Western medicine with Western medicine alone in 54 patients with DN. Acupuncture points included BL18, Weiwanxiashu (EX-B3), BL23, and CV4. The combined group showed significant reductions in total cholesterol (TC) and triglycerides (TG) and an increase in high-density lipoprotein (HDL), indicating that acupuncture with Western medicine can more rapidly improve lipid metabolism and help protect renal function. Tang et al. (74) randomly assigned 68 patients with DN of the spleen–kidney yang deficiency type to a Western medicine group or a warm needling plus Western medicine group. Warm needling was applied at BL23, BL20, Mingmen (GV4), ST36, SP6, Shenguan (GB3), CV6, and CV4. The combined group showed greater improvements in 24-h UP, Scr, serum albumin, and symptom scores, with a higher overall effective rate than the Western medicine group alone. Wang et al. (75) conducted a randomized controlled trial with over 70 patients with DN. In addition to standard treatment, the intervention group received spleen–stomach regulation acupuncture at Hegu (LI4), SP10, Quchi (LI11), CV12, Yinlingquan (SP9), and ST36. After treatment, the acupuncture group showed significantly lower levels of serum carbonyl products, malondialdehyde (MDA), and 8-hydroxydeoxyguanosine (8-OHdG), along with increased SOD activity, suggesting that acupuncture may enhance the renal protective effects of conventional therapy by reducing oxidative stress. Yu et al. (76) conducted a meta-analysis showing that acupuncture combined with Western medicine was superior to Western medicine alone, with higher overall efficacy (RR = 1.35), reduced proteinuria and Scr, improved HbA1c, glucose, and lipid profiles, and good safety. Data mining also identified commonly used effective acupoints (CV12, SP8, SP10, ST36, SP6, BL20, BL23, and SP9), providing a reference for standardized treatment.

In summary, acupuncture combined with Western medicine can improve proteinuria and renal function in a shorter time, with advantages in faster onset and better control of oxidative stress and early kidney injury markers. Compared with acupuncture alone, combined therapy places greater emphasis on integrating spleen–kidney regulation with metabolic control, and some protocols add abdominal Conception Vessel and Stomach Meridian points to enhance regulation of blood glucose, lipids, and oxidative stress. Most current studies are limited by small sample sizes, single-center designs, and insufficient safety reporting. Future research should not only confirm efficacy but also establish systematic safety monitoring to clarify the risk–benefit profile of this combined therapy.

### 3.5 Acupuncture combined with TCM and Western medicine

Gai et al. (77) randomly divided 98 patients with diabetic nephropathy (DN) into a Western medicine group (routine hypoglycemic and lipid-lowering therapy) and a traditional Chinese medicine (TCM) group (oral Yiqi Ruanjian Formula Decoction combined with acupuncture in addition to hypoglycemic and lipid-lowering therapy). The prescription in the TCM group included Huang Qi (Radix Astragali) 30 g, Bai Zhu (Rhizoma Atractylodis Macrocephalae) 15 g, Gui Jian Yu (Euonymus alatus) 20 g, Fu Ling (Poria) 15 g, Muxiang (Aucklandiae Radix) 15 g, Ge Gen (Radix Puerariae) 15 g, Dan Shen (Radix Salviae Miltiorrhizae) 10 g, and Chuan Xiong (Rhizoma Chuanxiong) 10 g. Acupoints were LI11, LI4, ST36, TE6, SP10, SP6, ST25, CV12, and CV3. The study found that integrated TCM and Western medicine significantly improved renal function, increased serum hepatocyte growth factor (HGF) levels, and decreased cystatin C and transforming growth factor-β1 (TGF-β1). Zhang et al. (78) recruited 76 patients with DN of the spleen-qi and kidney-qi deficiency pattern. The experimental group received a combination of Western medicine symptomatic treatment, acupuncture (acupoints: BL23, BL20, ST36, ST40, SP8, SP6, SP9, LI4, ST25, and CV12), and an invigorating spleen and kidney recipe [Huang Qi (Radix Astragali) 20 g, Shan Yao (Rhizoma Dioscoreae) 15 g, Shu Di (Rehmanniae Radix Praeparata) 10 g, Ze Xie (Alisma plantago) 10 g, Fu Ling (Poria) 10 g, Cang Zhu (Atractylodes lancea) 15 g, Qian Shi (Euryales Semen) 10 g, Sang Piao Xiao (Mantidis Ootheca) 10 g, Dan Shen (Radix Salviae Miltiorrhizae) 10 g, and Gan Cao (Radix Glycyrrhizae) 10 g]. These treatments significantly improved renal function and helped control blood glucose and lipid levels. Han et al. (79) selected 50 patients with DN and divided them into a control group and a treatment group according to a random-number table. Both groups received low-sodium, low-fat, high-quality protein diets and conventional Western medicine symptomatic treatment. The treatment group additionally received the Jieyu Jianpi Zishen Quyu Decoction [prescription: Chai Hu (Radix Bupleuri) 10 g, Curcuma aromatica Salisb. 10 g, Huang Qi (Radix Astragali) 30 g, Fu Ling (Poria) 20 g, Hou Po (Houpoea officinalis) 10 g, Bing Lang (Areca catechu L.), Shu Di (Rehmanniae Radix Praeparata) 15 g, Shan Yu Rou (Fructus Corni) 10 g, Fu Pen Zi (Rubus idaeus L.) 20 g, Bai Hua She She Cao (Scleromitrion diffusum) 25 g, Jiao Gu Lan (Gynostemma pentaphyllum) 10 g, Huang Lian (Rhizoma Coptidis) 6 g, Dan Shen (Radix Salviae Miltiorrhizae) 20 g, Di Long (Pheretima) 10 g, San Qi (Panax Notoginseng) 1.5 g, Da Huang (Radix et Rhizoma Rhei) 6 g, and Xi Huang Cao (Isodon serra) 15 g], together with acupuncture (acupoints: EX-B2, EX-B3, LR14, LR13, CV12, ST25, SP8, and KI3). The results showed that the combined treatment reduced blood glucose and lipid concentrations, improved renal function, and decreased urinary protein levels in patients.

In summary, acupuncture combined with integrated Chinese and Western medicine can improve proteinuria, renal function, and glucose and lipid metabolism in patients with DN. It also regulates fibrotic and reparative signals such as TGF-β1 and HGF, suggesting a potential role in delaying renal fibrosis. Core acupoints include ST36, SP6, and BL23, often used with CV12, CV3/CV4, SP10, ST40, and LI4 to balance spleen–kidney function and promote circulation, while herbal formulas focus on tonifying qi, strengthening the spleen, supporting the kidney, and activating blood flow. Compared with acupuncture alone or acupuncture combined with herbal medicine, this integrative approach provides broader regulation; however, current mechanistic evidence is mostly inferential and lacks validation from basic research. Limitations also include low methodological quality and short follow-up. Future studies should adopt integrated mechanism–clinical designs and include long-term follow-up to confirm its potential in antifibrosis and immune regulation (**Table 2**).

**Table 2:** Comparison of acupuncture alone, with herbal medicine, with Western medicine, and with triple therapy.

Treatment regimen	Typical acupoint combinations	Main mechanisms	Improved primary outcomes	Advantages	Limitations	Recommended population
Acupuncture alone	ST36、SP6、BL23 ± CV12/CV4/ST40/SP10	Root–branch coordination: anti-inflammatory, antioxidant, microcirculation & metabolism improvement	24h-UP、UAER/UACR↓	Good safety; holistic regulation; multi-target effects	Small samples; short follow-up; heterogeneous efficacy	Early DN; add-on/alternative for drug intolerance or contraindications
Acupuncture + Chinese medicine	ST36、SP6、BL23 (± CV12/SP10) Chinese medicine: Qi–spleen tonics; Kidney–blood activators; Dampness eliminators	Root–branch coordination; podocyte protection & autophagy; anti-inflammatory support	24 h-UP、UAER/UACR、Scr/Alb↓	Syndrome-specific; symptom & lab improvement	Uneven methodological quality; acupuncture vs. Chinese medicine effects hard to disentangle	Spleen–kidney deficiency, damp-heat with stasis; patients requiring symptom & metabolic improvement
Acupuncture + Western medicine	ST36, SP6, BL23 ± CV12, SP9, LI4, SP10 Standard therapy including hypoglycemic agents, RAAS blockade, SGLT2 inhibitors	Complementary to standard therapy: oxidative stress↓ (8-OHdG, MDA↓), lipid & microcirculation improvement, podocyte/endothelial protection	24 h-UP, Scr/BUN↓; lipid profile, HbA1c↓; faster onset	Guideline-compatible; early efficacy enhancement	Single-center, small samples; limited safety reports	Patients on Western medicine; need faster reduction of proteinuria/oxidative stress
Acupuncture + Chinese medicine + Western medicine	Combined with the above acupoint groups Chinese medicine: tonify spleen/kidney, activate blood, eliminate dampness; Western medicine: standard regimens	Broad-spectrum synergy: anti-inflammatory/antioxidant/metabolic + TGF-β1↓, CysC↓, HGF↑	24 h-UP、UAER/UACR、Scr/Alb↓、8-OHdG↓、SOD↑、TGF-β1、CysC↓	Broadest action; advantageous for complex phenotypes	Mechanisms mostly inferred; few multicenter RCTs & long-term follow-ups	Middle/late-stage or high risk of metabolic disorder/fibrosis; need multidimensional management

“↑” denotes an increase (higher value) and “↓” denotes a decrease (lower value) relative to the comparator (control/baseline).

**Figure 2:**
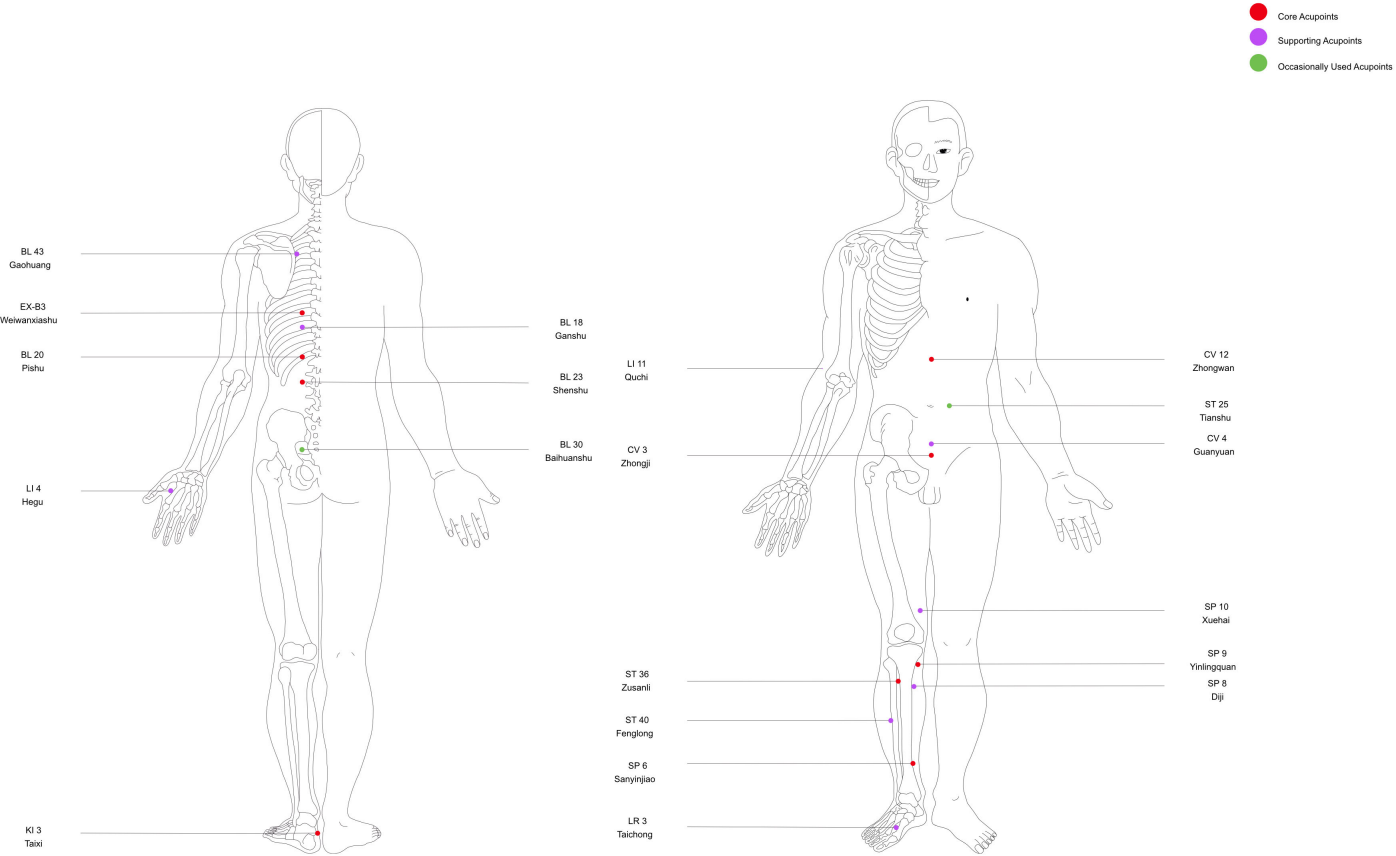
Distribution of acupuncture points used for diabetic nephropathy interventions. Schematic representation of commonly applied acupoints mapped on the human body (anterior and posterior views). Core acupoints (red), supporting acupoints (purple), and occasionally used acupoints (green) are highlighted.

To provide a clearer anatomical overview of the commonly used acupoints summarized above, a schematic diagram of their distribution on the anterior and posterior body surfaces is presented below (**Figure 2**).

## 4 Discussion and future directions

Current evidence shows consistent findings across outcomes, mechanisms, and prescriptions. Proteinuria-related indicators (24-h UP, UAER/UACR) improve most consistently (62, 65, 80), with some studies also reporting reduced Scr and BUN, while early-stage patients show increased eNOS/NO (81, 82). Mechanistically, acupuncture—especially “root–branch” prescriptions and core acupoints such as ST36, SP6, and BL23—can inhibit the HMGB1/NLRP3/NF-κB pathway, reduce oxidative stress, activate SIRT1/PGC-1α/FoxO1-PGC-1α signaling, and upregulate autophagy-related proteins, thereby supporting podocyte protection (33, 64, 65, 83). Compared with acupuncture alone, combinations with Chinese medicine or Western medicine provide broader and more consistent benefits, while triple therapy further regulates antifibrotic and reparative signals (TGF-β1↓, CysC↓, HGF↑), suggesting a potential therapeutic pathway from inflammation and oxidative stress toward fibrosis reversal and tissue repair (72, 84).

The contributions of this review are threefold. First, it identifies “common pathways” shared by Chinese and Western medicine—immune inflammation, oxidative stress, metabolic disorder, hemodynamic abnormality, and autophagy imbalance—and links molecular pathways (HMGB1/NLRP3/NF-κB, SIRT1/PGC-1α, PTEN/PI3K/Akt, autophagy and podocyte markers) with clinical outcomes (24-h UP, Scr/BUN, SOD/MDA, nephrin/podocin), building a testable pathogenesis–mechanism–indicator–prescription framework. Second, it compares intervention models (acupuncture alone, acupuncture with Chinese medicine, acupuncture with Western medicine, and triple therapy), summarizing core acupoint combinations and functional priorities to guide clinical choices. Third, it highlights the “root–branch acupoint” approach and frequent use of ST36, SP6, and BL23, with clear links to endothelial, antioxidative, mitochondrial–autophagy, and podocyte pathways, improving the review’s clinical relevance and verifiability.

Despite these promising findings, the current body of research still has clear limitations. Most studies are single-center, with small sample sizes and short follow-up periods, and reporting of randomization and blinding is often incomplete. Safety monitoring is inconsistent, adverse event reporting is insufficient, intervention models overlap, and outcome measures are heterogeneous, with core endpoints rarely assessed alongside mechanistic and patient-reported outcomes (36, 76, 85). Future research should conduct large, multicenter randomized controlled trials (RCTs) with rigorous design, standardized safety monitoring, and a core outcome set focused on 24-h UP, UAER/UACR, and eGFR slope, combined with mechanistic and patient-reported outcomes. Stratified or factorial designs are also needed to distinguish the independent and interactive effects of acupuncture and herbal medicine. Moreover, protocols based on ST36, SP6, and BL23 using “root–branch” acupoint strategies should integrate follow-up of mitochondrial–autophagy, podocyte, and endothelial markers to link mechanisms with clinical outcomes.

In addition to methodological concerns, it is also important to address treatment safety in a more systematic way. Across included clinical studies, acupuncture was generally well tolerated, with only minor adverse events such as transient needling pain or local bruising, and no serious adverse events were recorded (84, 86). However, reporting was inconsistent and follow-up windows were short, especially in combination regimens. This highlights the need for future studies to adopt standardized safety monitoring that includes hypoglycemia, infection, and drug–acupuncture interactions, in order to establish a more robust safety profile.

In clinical practice, strategies should be tailored to both evidence and patient characteristics. For early DN, acupuncture may serve as an adjunct to reduce proteinuria and improve microcirculation. Patients with high metabolic or oxidative stress may benefit more from combining acupuncture with Western medicine, while those with spleen–kidney deficiency and damp–heat with blood stasis may respond better to acupuncture plus Chinese medicine or triple therapy under careful monitoring. Prescriptions are usually based on ST36, SP6, and BL23, with CV12, CV4/CV6, ST40, SP10, LR3, and LI4 added to balance spleen–kidney function and improve circulation. Key markers such as eNOS/NO, SOD/MDA, HMGB1/NLRP3/NF-κB, SIRT1/PGC-1α, and nephrin/podocin should be tracked to link mechanisms with outcomes.

As a narrative review, systematic searching and formal risk-of-bias assessment were not performed. Most included studies had small samples, short follow-up periods, and heterogeneous endpoints, limiting overall evidence strength. Nevertheless, by integrating evidence from both Western medicine and TCM (2010–2025), this work proposes a pathogenesis–mechanism–indicator–prescription framework that provides a foundation and direction for future systematic reviews and meta-analyses conducted under PRISMA guidelines. Overall, this review consolidates fragmented findings into a practice-oriented framework, while underscoring the need for high-quality studies to further strengthen the evidence base and support the clinical application of acupuncture and its combined therapies in diabetic nephropathy.

## 5 Conclusions

Acupuncture—particularly centered on ST36, SP6, and BL23 with appropriate Biao–Ben (root–branch) acupoint combinations—shows potential in diabetic nephropathy by regulating inflammatory, metabolic, and antifibrotic pathways. Evidence suggests that acupuncture can reduce proteinuria, improve renal function, and enhance microcirculation, with broader effects when combined with Chinese or Western medicine. Triple therapy may provide the most comprehensive regulation, especially in progressive disease. These findings support the integration of acupuncture into multimodal management strategies for diabetic nephropathy. Future studies should adopt stratified clinical trial designs to validate stage- and pattern-specific applications.
